# Supplementary material for: Transcriptomics and Metabolomics Analysis of Sclerotium rolfsii Fermented with Differential Carbon Sources
Source: Foods. 2022 Nov 18;11(22):3706. doi: 10.3390/foods11223706 (PMC9689419; doi:10.3390/foods11223706)
Supplement: Supplementary file 1 [file foods-11-03706-s001.zip › foods-1992788-supplementary.pdf]

foods

MDPI

Supplementary Material

Table S1. Correlation analysis of DEGs and DEMs between SEPS_48 and GEPS_48 comparison groups.

Gene_Name	Metabolite_Name	Correlation	*p*-Value	q-Value
TRINITY_DN5421_c0_g1	Uridine 5′-diphosphate	1	0	0
TRINITY_DN5876_c0_g1		1	0	0
TRINITY_DN6972_c0_g3		1	0	0
TRINITY_DN7097_c0_g1		1	0	0
TRINITY_DN7130_c1_g2		1	0	0
TRINITY_DN7723_c1_g2		1	0	0
TRINITY_DN7956_c1_g2		1	0	0
TRINITY_DN8037_c2_g1		1	0	0
TRINITY_DN8142_c0_g1		1	0	0
TRINITY_DN8600_c3_g2		1	0	0
TRINITY_DN9347_c0_g2		1	0	0
TRINITY_DN9515_c0_g4		1	0	0
TRINITY_DN9516_c0_g1		1	0	0
TRINITY_DN9649_c7_g1		1	0	0
TRINITY_DN5237_c0_g1	L-Arginine	−1	0	0
TRINITY_DN6534_c0_g7		−1	0	0
TRINITY_DN6538_c1_g1		1	0	0
TRINITY_DN6689_c2_g2		−1	0	0
TRINITY_DN7657_c2_g1		−1	0	0
TRINITY_DN7685_c0_g2		1	0	0
TRINITY_DN8152_c1_g2		−1	0	0
TRINITY_DN8361_c0_g2		−1	0	0
TRINITY_DN8635_c1_g2		−1	0	0
TRINITY_DN8896_c0_g1		−1	0	0
TRINITY_DN3670_c0_g2	UDP-glucose	1	0	0
TRINITY_DN4084_c0_g1		1	0	0
TRINITY_DN5709_c0_g1		1	0	0
TRINITY_DN6365_c0_g3		1	0	0
TRINITY_DN7613_c0_g1		1	0	0
TRINITY_DN7777_c1_g2		1	0	0
TRINITY_DN7994_c0_g1		1	0	0
TRINITY_DN8363_c3_g1		1	0	0
TRINITY_DN8474_c1_g2		1	0	0
TRINITY_DN8492_c1_g1		1	0	0
TRINITY_DN8512_c0_g1		1	0	0
TRINITY_DN8525_c1_g1		1	0	0
TRINITY_DN8612_c0_g1		1	0	0
TRINITY_DN9111_c0_g3		1	0	0
TRINITY_DN9120_c0_g1		1	0	0
TRINITY_DN9562_c0_g1		1	0	0
TRINITY_DN5593_c0_g1	Hypoxanthine	1	0	0
TRINITY_DN6636_c0_g2		1	0	0
TRINITY_DN6938_c0_g1		1	0	0
TRINITY_DN7131_c0_g1		1	0	0

TRINITY_DN8716_c0_g2		−1	0	0
TRINITY_DN7934_c1_g1	Ribose 1-phosphate	1	0	0
TRINITY_DN7748_c0_g1		−1	0	0
TRINITY_DN8810_c0_g3		−1	0	0
TRINITY_DN9016_c0_g1	Uridine	−1	0	0
TRINITY_DN5593_c0_g1	Inosine	1	0	0
TRINITY_DN6636_c0_g2		1	0	0
TRINITY_DN6938_c0_g1		1	0	0
TRINITY_DN7131_c0_g1		−1	0	0
TRINITY_DN8716_c0_g2		−1	0	0
TRINITY_DN5105_c0_g1	UDP-D-apiose	−1	0	0
TRINITY_DN5808_c0_g1		1	0	0
TRINITY_DN8388_c0_g6		−1	0	0
TRINITY_DN9088_c0_g1		1	0	0

Table S2. Number of pathways annotated by DEGs and DEMs.

Second Category	Pathway ID	Pathway Description	Metabolite Number	Gene Number
Nucleotide metabolism	map00230	Purine metabolism	8	78
Carbohydrate metabolism	map00520	Amino sugar and nucleotide sugar metabolism	8	63
Membrane transport	map02010	ABC transporters	7	8
Lipid metabolism	map00564	Glycerophospholipid metabolism	7	42
Carbohydrate metabolism	map00052	Galactose metabolism	7	28
Global and overview maps	map01210	2-Oxocarboxylic acid metabolism	6	0
Carbohydrate metabolism	map00051	Fructose and mannose metabolism	6	26
Global and overview maps	map01200	Carbon metabolism	5	0
Amino acid metabolism	map00330	Arginine and proline metabolism	5	52
Nucleotide metabolism	map00240	Pyrimidine metabolism	5	56
Carbohydrate metabolism	map00040	Pentose and glucuronate interconversions	4	32
Amino acid metabolism	map00220	Arginine biosynthesis	4	20
Amino acid metabolism	map00250	Alanine, aspartate and glutamate metabolism	4	31
Metabolism of other amino acids	map00410	beta-Alanine metabolism	4	20
Carbohydrate metabolism	map00630	Glyoxylate and dicarboxylate metabolism	4	37
Metabolism of cofactors and vitamins	map00770	Pantothenate and CoA biosynthesis	4	10
Metabolism of other amino acids	map00480	Glutathione metabolism	4	36
Biosynthesis of other secondary metabolites	map00332	Carbapenem biosynthesis	3	2
Carbohydrate metabolism	map00020	Citrate cycle (TCA cycle)	3	38
Lipid metabolism	map00590	Arachidonic acid metabolism	2	18
Metabolism of other amino acids	map00472	D-Arginine and D-ornithine metabolism	2	3

Metabolism of cofactors and vitamins	map00860	Porphyrin and chlorophyll metabolism	2	22
Amino acid metabolism	map00340	Histidine metabolism	2	32
Energy metabolism	map00190	Oxidative phosphorylation	2	121
Lipid metabolism	map00561	Glycerolipid metabolism	2	32
Carbohydrate metabolism	map00562	Inositol phosphate metabolism	2	16
Carbohydrate metabolism	map00053	Ascorbate and aldarate metabolism	2	17
Amino acid metabolism	map00360	Phenylalanine metabolism	2	36
Carbohydrate metabolism	map00660	C5-Branched dibasic acid metabolism	2	1
Carbohydrate metabolism	map00030	Pentose phosphate pathway	2	24
Metabolism of cofactors and vitamins	map00740	Riboflavin metabolism	2	8
Energy metabolism	map00680	Methane metabolism	2	24
Metabolism of other amino acids	map00460	Cyanoamino acid metabolism	2	15
Metabolism of cofactors and vitamins	map00730	Thiamine metabolism	2	11
Carbohydrate metabolism	map00500	Starch and sucrose metabolism	2	46
Translation	map00970	Aminoacyl-tRNA biosynthesis	2	20
Lipid metabolism	map00073	Cutin, suberine and wax biosynthesis	1	0
Biosynthesis of other secondary metabolites	map00232	Caffeine metabolism	1	0
Drug resistance: Antimicrobial	map01502	Vancomycin resistance	1	0
Transport and catabolism	map04136	Autophagy–other	1	0
Amino acid metabolism	map00310	Lysine degradation	1	24
Biosynthesis of other secondary metabolites	map00311	Penicillin and cephalosporin biosynthesis	1	3
Amino acid metabolism	map00400	Phenylalanine, tyrosine and tryptophan biosynthesis	1	21
Lipid metabolism	map00061	Fatty acid biosynthesis	1	15
Lipid metabolism	map00100	Steroid biosynthesis	1	24
Metabolism of cofactors and vitamins	map00750	Vitamin B6 metabolism	1	6
Signal transduction	map04070	Phosphatidylinositol signaling system	1	16
Metabolism of other amino acids	map00430	Taurine and hypotaurine metabolism	1	9
Lipid metabolism	map00592	alpha-Linolenic acid metabolism	1	7

Table S3. Transcriptomics expression analysis.

**Gene_ID**	**FC(SEPS/GEPS)**	**KO_Name**	**Pathway_ID**	***p*-Value**
TRINITY_DN8531_c2_g6	1.243	POLE4	map00240	0.000190911
TRINITY_DN248_c0_g1	1.212	RPB5, POLR2E		2.65×10^{-14}
TRINITY_DN4569_c0_g2	0.817	RPB12, POLR2K		2.26×10^{-8}
TRINITY_DN9553_c0_g1	0.795	K16330		0.002307208
TRINITY_DN7584_c1_g1	0.793	codA		0.001593013
TRINITY_DN1642_c0_g1	0.784	carB, CPA2		2.25×10^{-21}
TRINITY_DN4905_c0_g1	0.578	pyrD		0.00272933
TRINITY_DN9342_c0_g1	0.045	CANT1		0.011851808
TRINITY_DN8267_c0_g2	0.023	punA, PNP		0.000154903
TRINITY_DN11464_c0_g1	0.004	ndk, NME		7.90×10^{-11}

TRINITY_DN7350_c0_g1	1.919	POLD3		0.00674508
TRINITY_DN7695_c0_g2	0.829	AMPD		6.56×10^{-5}
TRINITY_DN5313_c0_g1	0.815	nudF		0.000157083
TRINITY_DN3898_c0_g2	0.812	cysC		4.59×10^{-8}
TRINITY_DN8239_c0_g1	0.803	add, ADA		0.006654052
TRINITY_DN6528_c0_g1	0.763	uaZ		5.33×10^{-40}
TRINITY_DN11275_c0_g1	0.755	guaA, GMPS		1.04×10^{-15}
TRINITY_DN2888_c0_g2	0.754	FHIT	map00230	1.31×10^{-9}
TRINITY_DN7903_c1_g2	0.73	POLD4		0.003086768
TRINITY_DN9751_c0_g1	0.706	CECR1, ADA2		6.02×10^{-7}
TRINITY_DN9217_c0_g2	0.645	POLD3		0.007793758
TRINITY_DN11405_c0_g1	0.627	PRUNE, PPX1		4.63×10^{-8}
TRINITY_DN5896_c0_g3	0.6	alc, ALLC		1.74×10^{-12}
TRINITY_DN2786_c0_g2	0.599	APRT, apt		7.95×10^{-8}
TRINITY_DN4445_c0_g2	0.041	GUCY2C		0.00682107
TRINITY_DN3430_c0_g3	0.004	add, ADA		1.06×10^{-11}
TRINITY_DN2971_c0_g1	0.784	proB		0.000724858
TRINITY_DN9859_c0_g1	0.675	pip		0.003788367
TRINITY_DN4066_c0_g1	0.661	E4.3.1.12, ocd	map00330	1.73×10^{-7}
TRINITY_DN543_c0_g1	0.036	E2.7.3.2		1.47×10^{-9}
TRINITY_DN1814_c0_g1	0.003	E2.7.3.2		9.75×10^{-12}
TRINITY_DN6483_c1_g10	0.003	GAMT		1.01×10^{-11}
TRINITY_DN4575_c0_g1	1.414	GOT1		5.36×10^{-13}
TRINITY_DN6741_c1_g2	1.31	GPT, ALT		2.01×10^{-8}
TRINITY_DN9515_c0_g4	1.295	ARG56		5.74×10^{-114}
TRINITY_DN10996_c0_g1	0.77	GDH2		0.005099424
TRINITY_DN9626_c1_g1	0.743	URE		0.003233872
TRINITY_DN8000_c0_g1	0.742	argH, ASL	map00220	0.000282961
TRINITY_DN4450_c0_g1	0.656	E1.4.1.4, gdhA		1.30×10^{-6}
TRINITY_DN2449_c0_g1	0.038	GOT2		2.53×10^{-15}
TRINITY_DN7210_c0_g3	0.036	GOT1		5.36×10^{-13}
TRINITY_DN5004_c0_g1	0.024	GOT1		2.01×10^{-8}
TRINITY_DN11101_c0_g1	0.012	GOT2		5.74×10^{-114}
TRINITY_DN3271_c0_g1	0.001	GLUD1_2, gdhA		0.005099424
TRINITY_DN3772_c0_g1	1.208	DAO, aao	map00472	2.62×10^{-8}
TRINITY_DN3642_c0_g1	0.018	DAO, aao		3.18×10^{-5}
TRINITY_DN6723_c2_g2	1.601	UAP1		2.97×10^{-202}
TRINITY_DN9329_c0_g2	1.399	PGM3		4.02×10^{-29}
TRINITY_DN7104_c0_g1	1.367	CHS1		1.13×10^{-5}
TRINITY_DN7044_c0_g1	1.344	manC, cpsB		6.83×10^{-73}
TRINITY_DN7442_c0_g2	1.328	glmS, GFPT		2.67×10^{-145}
TRINITY_DN8273_c0_g4	1.323	CHS1		5.86×10^{-7}
TRINITY_DN7393_c0_g1	1.29	CHS1		1.32×10^{-98}
TRINITY_DN224_c0_g1	1.244	CHS1	map00520	7.02×10^{-58}
TRINITY_DN7439_c1_g1	1.228	E3.2.1.14		1.59×10^{-15}
TRINITY_DN4660_c0_g2	1.228	CHS1		0.007327476
TRINITY_DN675_c0_g1	1.223	CHS1		2.11×10^{-54}
TRINITY_DN5865_c1_g1	1.209	CHS1		1.24×10^{-48}
TRINITY_DN6701_c0_g1	1.203	CHS1		1.09×10^{-10}
TRINITY_DN9418_c0_g1	1.203	manA, MPI		2.79×10^{-9}
TRINITY_DN7657_c3_g5	0.825	E3.2.1.14		4.48×10^{-7}

TRINITY_DN8159_c0_g1	0.729	nagB, GNPDA	4.32×10^{-15}
TRINITY_DN3475_c1_g1	0.707	PMM	2.96×10^{-5}
TRINITY_DN3810_c0_g1	0.031	UGP2, galU, galF	0.001275142
TRINITY_DN8094_c0_g1	0.015	nagA, AMDHD2	1.00×10^{-5}
TRINITY_DN4707_c0_g1	0.007	E1.6.2.2	1.66×10^{-8}
TRINITY_DN1701_c0_g1	0.004	UGDH, ugd	4.47×10^{-11}
TRINITY_DN6645_c0_g1	0	E3.2.1.14	2.50×10^{-49}

Table S4. Metabolomics expression analysis.

Metabolite	Metab ID	VIP_pred_OPLS-DA	FC(SEPS_48/GEPS_48)	*p*-Value	KEGG Pathway ID
Uridine 5′-monophosphate	metab_6863	1.930936874	1.052474171	0.003431	map00240;map01100
5′-CMP	metab_11041	2.057205287	1.042540793	0.003371	map00240;map01100
Uridine 5′-diphosphate	metab_70	1.362161685	1.024282561	0.004412	map00240;map01100
UDP-glucose	metab_10933	1.619704427	1.019764635	0.004536	map01100;map00561;map00520;map00040;map00053;map00052;map00240;map00500;map01130
Cytosine	metab_1387	1.001835968	0.98066359	0.04633	map00240;map01100
Uridine	metab_5872	1.530457094	0.973458488	0.03786	map00240;map01100
Thymidine	metab_5132	4.46137787	0.680851064	1.08E−05	map00240;map01100
Guanine	metab_1329	2.323581715	1.064842356	0.000247	map01100;map00230
Adenosine diphosphate ribose	metab_10838	1.579882855	1.031841831	0.0399	map00230
Ribose 1-phosphate	metab_5857	1.427342114	0.980786687	0.01462	map00030;map00230
Xanthine	metab_10826	2.168029578	0.95200537	0.01182	map01100;map01110;map00230;map00232
Hypoxanthine	metab_1327	1.978168206	0.950570342	0.005911	map01100;map00230
Inosine	metab_6079	2.594051296	0.936148819	0.008059	map01100;map00230
Adenine	metab_10778	2.798507694	0.923167385	0.002147	map01100;map00230
Adenosine	metab_5866	3.120938255	0.909124356	0.002698	map01100;map00230
Xanthosine	metab_10656	2.922169492	0.908485118	0.000846	map01100;map01110;map00230;map00232
Putrescine	metab_5706	1.491136371	1.047640551	0.04606	map01100;map00480;map01110;map02010;map00330;map01130
L-Proline	metab_5484	1.295335324	0.981170141	0.003943	map01100;map01110;map02010;map00330;map01230;map00332;map00970;map01130;map00404
L-Arginine	metab_5750	1.840548692	0.966525837	0.0004915	map01100;map00220;map01110;map00472;map02010;map00330;map01230;map00970;map00261;map01130
Argininosuccinic acid	metab_5549	1.444163315	0.967115427	0.005936	map01100;map00220;map01110;map01230;map00250;map01130
D-Ornithine	metab_281	1.229394458	0.971525424	0.03692	map01100;map00472
UDP-D-apiose	metab_10927	1.695995011	1.039862404	0.03918	map00520
Dulcitol	metab_11044	3.883634915	1.152235966	4.44E−06	map01100;map00052

D-Sorbitol	metab_5511	2.77653723	1.1	7.38E−05	map01100;map02010;map00051;map00052
12-OPDA	metab_4691	2.02519197	1.044491154	0.0002434	map01100;map00592;map01110
(2′E,4′Z,7′Z,8E)-Colnelenic acid	metab_4260	1.064861987	1.017294389	0.01741	map00592;map01110
PC(14:0/20:3(8Z,11Z,14Z))	metab_634	1.116831137	0.983058539	0.04532	map01100;map00564;map01110;map00590;map00591;map00592
